# Genetic and Molecular Heterogeneity of Synovial Sarcoma and Associated Challenges in Therapy

**DOI:** 10.3390/cells13201695

**Published:** 2024-10-14

**Authors:** Ekaterina A. Lesovaya, Timur I. Fetisov, Beniamin Yu. Bokhyan, Varvara P. Maksimova, Evgeny P. Kulikov, Gennady A. Belitsky, Kirill I. Kirsanov, Marianna G. Yakubovskaya

**Affiliations:** 1 Department of Chemical Carcinogenesis, N.N. Blokhin National Medical Research Center of Oncology, Ministry of Health of Russia, Moscow 115478, Russia; lesovenok@yandex.ru (E.A.L.); timkatryam@yandex.ru (T.I.F.); beniamin-bokhyan@mail.ru (B.Y.B.); lavarvar@gmail.com (V.P.M.); belitsga@mail.ru (G.A.B.); kkirsanov85@yandex.ru (K.I.K.); 2 Oncology Department, Ryazan State Medical University Named after Academician I.P. Pavlov, Ministry of Health of Russia, Ryazan 390026, Russia; e.kulikov@rzgmu.ru; 3 Institute of Medicine, RUDN University, Moscow 117198, Russia

**Keywords:** synovial sarcoma, chromosomal translocation, SS18-SSX, epigenetic changes, T-cell receptor gene therapy, targeted therapy, tyrosine kinase inhibitors

## Abstract

Synovial sarcoma (SS) is one of the most common types of pediatric soft tissue sarcoma (STS) being far less frequent in adults. This STS type is characterized by one specific chromosomal translocation SS18-SSX and the associated changes in signaling. However, other genetic and epigenetic abnormalities in SS do not necessarily include SS18-SSX-related events, but abnormalities are more sporadic and do not correlate well with the prognosis and response to therapy. Currently, targeted therapy for synovial sarcoma includes a limited range of drugs, and surgical resection is the mainstay treatment for localized cancer with adjuvant or neoadjuvant chemotherapy and radiotherapy. Understanding the molecular characteristics of synovial sarcoma subtypes is becoming increasingly important for detecting new potential targets and developing innovative therapies. Novel approaches to treating synovial sarcoma include immune-based therapies (such as TCR-T cell therapy to NY-ESO-1, MAGE4, PRAME or using immune checkpoint inhibitors), epigenetic modifiers (HDAC inhibitors, EZH2 inhibitors, BRD disruptors), as well as novel or repurposed receptor tyrosine kinase inhibitors. In the presented review, we aimed to summarize the genetic and epigenetic landscape of SS as well as to find out the potential niches for the development of novel diagnostics and therapies.

## 1. Introduction

Synovial sarcoma (SS) accounts for 4–10% of all soft tissue sarcoma (STS) subtypes, being the fourth most frequent cancer after liposarcoma (26%), leiomyosarcoma (14%) and non-differentiated pleomorphic sarcoma (14%) [1,2]. SS is most frequently diagnosed in adolescents and young adults (aged 15–35) and is more prevalent in males; 80% of SS is localized in soft tissues of extremities. These malignancies are less frequently found in the head and neck, in the abdomen, joints, the mediastinum, the peripheral nerves, skin, and visceral organs [3,4,5,6].

The WHO defines SS as a mesenchymal spindle cell tumor with the possible presence of a tissue component with epithelial differentiation, including the formation of glandular structures, and with the specific pathognomonic reciprocal t(X;18) translocation, which causes the binding of SS18 with a homologous SSX gene (mainly SSX1 or SSX2, or SSX4 in rare cases). As a result, a chimeric SS18-SSX fusion protein is formed [3,4,6,7,8,9]. It is not accurate to use “synovial” in synovial sarcoma, as the development of the tumor is not limited to synovium and can start in any part of the body. Primary tumors developing from the synovial membrane are rare, but other mesenchymal tissue tumors can appear within the synovium. These tumors include synovial nodular fasciitis, synovial chondromatosis, synovial lipoma, synovial hemangioma, and synovial chondrosarcoma. These tumors are classified as poor-prognosis metastatic cancers [10].

The morphological variation within the same tumor can range from low grade to high grade. Moreover, a tumor that is originally of low-grade morphology may subsequently develop a more aggressive, high-grade phenotype [3,4,5]. In addition, the WHO classifies less aggressive benign and intermediate-grade tumors into separate groups. The growth of intermediate-grade tumors may cause infiltration and disruption, making their total resection challenging. These malignancies are locally aggressive and rarely metastasize (the metastatic potential is approximately 2%). Benign neoplasms are local and can be easily eliminated by surgery when possible. They do not grow in a disruptive way and do not possess metastatic potential.

The term “synovial sarcoma” was coined in a study by Simon et al. in 1895 and used to describe the malignant neoplasm of a knee joint in an adult male patient. This neoplasm was characterized in detail 15 years later [11]. The typical SS consists of spindle cell tissue with glandular-like structures, but the histological characteristics of SS may vary and include nuclei polarization, Homer–Wright pseudorosettes, massive foci of metaplastic bone and cartilage formation, and myxoid stroma [12,13,14]. The morphological variety of SS is evidenced by its multiple subtypes. The WHO classifies SS into the two main histological subtypes: monophasic spindle cells and biphasic ones, which account for more than 60% of total SS cases. Several classifications include the monophasic epithelial subtype, cystic, calcifying and myxoid histological subtypes [4]. Due to the remarkable variety of the tissues involved, SS verification includes a broad range of diagnostic techniques: MPNST, Ewing’s sarcoma/PNET, fibrosarcoma, leiomyosarcoma, epithelioid sarcoma, clear cell sarcoma, a diffuse-type tenosynovial giant cell tumor, palmar/plantar fibromatosis, hemangiopericytoma, sarcomatoid carcinoma, carcinosarcoma, and mesothelioma.

Most SS are highly malignant tumors with the risk of metastasis to lungs, lymph nodes, bones and the pleura, and the 5-year survival rate is less than 50% [15]. The difficulty in diagnosis at early stages is associated with numerous changes in signaling driven by characteristic SS18-SSX chromosomal translocation. It leads to the need for a precise description of the molecular status of the patients, the proper diagnostics and further rational planning of therapy. Multiple factors should be taken into account for precise diagnosis and personification of the treatment. Important pathological changes in SS are described below.

## 2. Genetic Abnormalities of SS

In total, 95% of SS are characterized by chromosomal translocation t(X:18)(p11.2:q11.2), which results in multiple chimeric gene variants: SS18-SSX1, SS18-SSX2 and SS18-SS4 [16]. The prognostic value of each chimeric gene differs depending on the gene type: SS18-SSX1 is associated with higher mortality and metastasis incidence than SS18-SSX2, but SS18-SSX2-positive patients more frequently develop a recurrent stage of the disease [14,16,17]. In addition to the well-known SS-specific translocation, in several cases, SS samples harboring the novel EWSR1-SSX1 and MN1-SSX1 fusions were described [18,19].

Apart from this well-known signature chromosomal translocation of SS, novel genetic abnormalities were described for this pathology, but their frequency may vary. Novel mutations in multidrug resistance genes from the ABC transporters family were described in the study based on 11 samples from SS patients, but the frequency was rather low [20]. Rare mutations in TP53 (less than 1%), as well as alterations within AKT/PTEN, CTNNB1, and APC, were identified, and the AKT/mTOR and Wnt signaling pathways were frequently activated [21]. Overall, the SS karyotype is generally simple and stable with few secondary genetic alterations. A retrospective analysis of SS at a low differentiation stage which harbors both SS18-SSX2 fusion and MDM2 amplification also revealed the low rate of double anomaly [22]. 

Overexpression of specific genes is more common for SS. The increased expression of CDCA2, KIF14, IGFBP7, Secernin-1, E2H2, MMPs, and CXCR4 genes is currently studied as a potential therapeutic target or a diagnostic tool [23]. The expression of transcriptional repressor GATA binding 1 (TRPS1) is frequent in synovial sarcoma, which is linked to the activity of the SS18-SSX fusion oncoprotein [24]. Studying the molecular mechanisms of BCL2, a negative regulator of apoptosis, in 32 SS patients revealed that it was expressed in 100% of cases. Therefore, antisense oligodeoxynucleotide targeted at BCL-2 (G3139) may serve as an appropriate therapeutic alternative for patients with BCL-2-positive synovial sarcomas [25]. Overexpression of the FOXM1 gene encoding a FOX protein was studied in 106 clinical samples of SS and correlates with a higher malignization rate confirmed by in vitro experiments [26]. In a study of 206 SS samples from patients, E3 ubiquitin ligase F-box protein SKP2 was overexpressed and associated with poor prognosis [27]. A recent study of 13 SS samples demonstrated that overexpression of SOX-2, PAX-7, INI-1 and NKX3.1 can be used as an ancillary tool [18,28].

The data based on 12 SS histological samples revealed several mutated genes related to the inflammatory response, adhesion, ECM–receptor interaction pathways, both TGF-β and JAK–STAT signaling, phenylalanine metabolism, the fibrin (neuroguidin (NGDN) intrinsic pathway and formation, RAS protein activator like 3 (RASAL3), KLHL34, MUM1L1, etc.) [29]. Identifying the inflammatory pathway activation in SS led to the discovery of upregulated oncostatin M receptor (OSMR) and its role in SS viability in vitro and in vivo [30]. The study of seven clinical SS samples demonstrated the increased expression of the EPHB4 gene encoding an Eph tyrosine kinase receptor family member. In addition, the downregulation of EPHB4 with RNA interference in vitro led to a decrease in the viability of SS cells [31]. The upregulation of the hepatocyte growth factor (HGF)/c-MET signaling pathway in SS was demonstrated in vitro and confirmed in patient samples suggesting the therapeutic potential of HGF inhibitors [32].

Synovial sarcoma expresses multiple cancer-testis antigens (CTAs), in particular, the preferentially expressed antigen in melanoma (PRAME) gene that could potentially be targeted by T-cell receptor (TCR) gene therapy. PRAME-expressing SS cells were efficiently recognized by PRAME-specific T-cells in vitro. PRAME expression was detected in all synovial sarcoma samples. An additional potential marker for successful PRAME-TCR-gene therapy is represented by an elevated level of HLA-I in highly malignant areas and areas infiltrated with T-cells in predominantly biphasic SS [33]. The high expression of New York esophageal squamous cell carcinoma 1 (NY-ESO-1) or the melanoma-associated antigen A4 (MAGE-A4) is also common for SS, and these results may be helpful for a synovial sarcoma diagnosis [21,34,35].

Analyzing the expression profile data (GSE40021) on SS metastasis from the Gene Expression Omnibus (GEO) database identified five hub genes (HJURP, NCAPG, TPX2, CENPA, NDC80) with differential expression in metastatic SS [36].

## 3. SS Epigenetic Features

SS pathogenesis is mainly driven by SS18-SSX fusion proteins playing an important role in the regulation of chromatin remodeling. The key epigenetic regulators, switch/sucrose-non-fermentable (SWI/SNF) complexes, are the members of the Trithorax-group protein (TrxG) family [37,38]. In general, there are three mammalian SWI/SNF chromatin remodeling complexes, SS18 is included in one of them, the canonical BRG1/BRM-associated factor (cBAF) complex. This complex was demonstrated to interact with the SS fusion proteins with a complete replacement of the wild-type SS18 in the cBAF complexes [37]. This leads to ejecting SWI/SNF-related matrix-associated actin-dependent regulators of the chromatin subfamily B member 1 (SMARCB1), which is subsequently degraded in proteasomes [9,37,39]. Polycomb repressive complexes (PRCs) are another epigenetic regulator family [38,39]. While PRCs are responsible for chromatin compaction and gene repression, SWI/SNF complexes facilitate transcription by nucleosome remodeling after gene activation via recruiting transcription factors to their binding sites [40]. The oncogenic SWI/SNF complexes could target PRC-repressed domains and activate them by recruiting RNA Polymerase II and initiating transcription. The SS18-SSX oncoprotein also interacts with PRC1 and PRC2 by colocalization with these complexes mediating transcriptional silencing [37]. 

For several heterogeneous non-canonical PRC1 complexes, the SS18-SSX protein was shown to utilize lysine demethylase 2B (KDM2B) as part of recruiting a non-canonical PRC1 (PRC1.1) to target cBAF at the unmethylated CpG islands. Therefore, BAF-mediated PRC2 antagonism and aberrant gene activation occur at these sites [38]. PRC2 performs chromatin silencing via its catalytic subunit Enhancer of Zeste 2 (EZH2), histone methyltransferase expressed in 76% of human synovial sarcoma samples. Furthermore, in vitro experiments demonstrated that EZH2 inhibition suppressed both cell growth and migration [41]. 

The above epigenetic changes result in multiple dysregulations of the signaling pathway. SWI/SNF-BAF chromatin remodeling and Polycomb repressive complexes epigenetically activate FGF receptor (FGFR) signaling. Two ETS factors and FGFR targets, ETS-related transcription factors Etv4 and Etv5, further drive synovial sarcoma growth via cell cycle regulation. Thus, they are potential biomarkers of SS pathogenesis [42].

Other SS18-SSX-related epigenetic changes described in the literature include the repression of the early growth response 1 (EGR1) by the SS18-SSX protein via associating with its promoter. SS18-SSX binding occurs via monomethylated, dimethylated, and trimethylated histone H3 Lys27 (H3K27me1, H3K27me2, and H3K27me3) and recruitment of Polycomb group proteins to this promoter. Furthermore, in vitro treatment of synovial sarcoma cells with the HDAC inhibitor romidepsin suppresses this modification and reactivates EGR1 expression [28,43,44,45]. Histone modifications H2AK118ub and H2AK119Ub (histone H2A mono-ubiquitination at lysine 119) were reported for SS18–SSX binding at H2AK119Ub regions as a component of SS pathogenesis [43,46]. The SS18 protein also affects histone modification through interactions with the AF10, p300, and SIN3A proteins [21]. In the study of five SS samples, the methylation profile showed differentially methylated regions and WT1-AS and TNXB genes were detected, which had not previously been affected in SS progression [47].

The changes in the level of several miRNAs are also reported for synovial sarcoma. Serum miR-92b-3p levels were significantly higher in two independent cohorts of SS patients in comparison with healthy volunteers, as well as other STS patients. Therefore, miR-92b-3p expression in the serum could be used for monitoring tumor dynamics of SS [48]. MiR-9, the miRNA promoting epithelial–mesenchymal transition and tumor metastasis, was demonstrated to promote tumorigenesis of SS in vitro. However, its specific overexpression in SS patients was not described [49]. Significant upregulation of twenty-one different microRNAs (including let-7e, miR-99b, and miR-125a-3p) was observed in SS, suggesting that these molecules also play a potential role in carcinogenesis [50].

## 4. Changes in SS Signaling Pathways and Corresponding Approaches to Therapy

Direct changes in the pathological signaling pathways in SS are associated with SS18-SSX fusion products and their interactors. Several SS18 targets, specifically BMI1 and RING1, which are transcriptional repressors from the PcG protein family, are described in the previous section. The protein–protein interactions between SS18-SSX and the transcriptional repressors from the PcG protein family lead to disbalance in BAF complexes, in particular, the bromodomain-containing protein 9 (BRD9) [51].

In addition, the SS18-SSX fusion protein is heavily involved in transcription regulation, protein transport and cell adhesion. The most well-known interactors of SS18-SSX are given in Table 1.

In addition to the above-mentioned direct targets, SS18-SSX oncoproteins indirectly regulate cell growth and proliferation via upregulation of cyclin D1, Wnt/β-catenin pathway components (increased phosphorylation and hyperactivation of AXIN2, CDC25A, c-MYC, DKK1, CyclinD1, Survivin, LEF1, TCF7, ZIC2, WNT5A, and FZD10), and TP53 pathway components [38,54,55], EGR1, insulin-like growth factor 2 (IGF-2) with its receptor IGF-1R [56]. The TLE1 gene (9q21.32) plays an independent role. This gene is a member of the TLE gene family encoding Groucho-like transcriptional corepressors, frequently overexpressed in SS [57]. It binds other basic helix–loop–helix proteins to repress the target genes of the Wnt/β-catenin signaling pathway [38]. 

Other genes and pathways with perturbations in SS but not directly associated with SS18-SSX, include the well-known cancer-related signaling pathways, namely Hedgehog (SMO, PTCH1), NY-ESO-1 (CTAG1A), Notch (JAG1, JAG2, and HES1), RTKs (FGF2, FGF3, EGFR, PDGFR, TGF, IGF and IGFBP), HGF/c-MET signaling components including changes in ALK and MET signaling phosphorylation patterns, GSK3β (phosphorylated), the DDR-related kinase ATR, regulators of cell motility, invasion and metastasis MMP-14, E-cadherin, N-cadherin, vimentin, and components of the TGF-β1/Smad, PI3K/Akt, MAPK/Erk signaling pathways. The described molecules include proproliferative and proinflammatory signaling, stimulators of neoangiogenesis, immune response as well as cell invasion and migration, and could serve as targets in the therapy [32,58,59,60,61,62,63,64,65,66,67]. 

There are also the YAP/TAZ transcriptional coactivators shuttling between the cytoplasm and the nucleus. They interact with other transcription factors, such as TEA family members (TEAD). The YAP/TAZ/TEAD bind to distant enhancers contacting the regulated promoters of their targets through DNA looping and promoting enhancer acetylation and recruitment of the transcriptional machinery thus controlling the expression of their targets [68]. The YAP/TAZ cause aberrant cell proliferation. For instance, the SS18-SSX-dependent activation of YAP/TAZ signals is a common pattern in SS [69]. In addition, mutual activation of the nuclear YAP1/TAZ-TEAD and b-catenin-TCF pathways in SS was demonstrated to interact with SS18-SSX, which is crucial for the interdependence of YAP1 and b-catenin in synovial sarcoma [70]. CDK9, a protein regulating RNA transcription, cell apoptosis and motility, occurs mainly in the cell nucleus and has high expression in human synovial sarcoma cell lines and in over 90% of human sarcoma tissue microarray samples. The highly expressed CDK was associated with a poor prognosis of SS patients and could be a promising treatment target [59]. The cAMP response element binding protein (CREB), the ubiquitously expressed transcription factor, was shown to be phosphorylated and activated in SS both in vitro and in vivo. CREB-mediated signals disrupted by small molecule inhibitors could be effective for SS treatment [71]. The EPH/ephrin signaling pathway regulating many developmental processes and adult tissue homeostasis is also aberrant in SS. The inhibition of EphB2 signaling in vitro was shown to reverse the aberrant cytoskeletal phenotype [72]. 

Despite significant advances in the molecular studies of the pathogenesis and progression of SS, surgery combined with neoadjuvant or adjuvant therapies (radiotherapy and chemotherapy) is the standard of care for this cancer type. The front-line therapy (anthracyclines and ifosfamide) is followed by the not-so-well-established second line of the minor groove binder trabectedin and receptor tyrosine kinase inhibitors such as pazopanib, sorafenib, and the monoclonal anti-VEGF antibody bevacizumab [15,73,74,75,76]. Ongoing clinical trials of innovative therapies for synovial sarcoma include several classes of low-molecular-weight compounds and monoclonal antibodies: 

Immune checkpoint inhibitors (anti-CTLA-4, anti-PD-1, anti-PD-L1) (successfully entered CT, the anti-PD-L1 antibody atezolizumab [77,78], the anti-PDL1 antibody durvalumab and the anti-CTLA-4 antibody tremelimumab, without marked efficacy in Phase II clinical trial [79], the anti-CTLA4 antibody ipilimumab and the antiPD-1 antibody nivolumab, which, in contrast, induced the best response in the SS cohort of patients [80]).

Receptor tyrosine kinase inhibitors (the multikinase VEGFR/PDGFR inhibitor regorafenib [81,82], the multikinase VEGFR/PDGFR inhibitor anlotinib [83]).

Epigenetic modifiers (HDAC inhibitors (romidepsin, entinostat, vorinostat) [15,74], EZH2 inhibitors [84], BDR9 degraders [15,74]).

The expression of cancer-testis antigens (CTAs), constituting a large family of antigens with elevated expression in the testes and in transformed cancer cells rather than in normal somatic cells, opens up new avenues for effective SS treatment. To specifically target CTA, including NY-ESO-1, or MAGE-A4, or PRAME, adoptive transfer of antigen-specific T-cell receptor-transduced T (TCR-T)-cells has shown promise for the metastatic SS treatment in several early phase clinical trials: efficacy was reached along with manageable safety profile (the most common adverse events were cytopenias in 25–75% cases) [15,74]. The results of SS-related clinical trials are given in Table 2.

Numerous novel targets for SS treatment are undergoing preclinical trials. TAS-115, a novel MET/VEGFR inhibitor, was demonstrated to suppress the activity of multiple RTKs and affect the viability of SS cells [85]. YM155 or Sepantronium bromide, a suppressant of the anti-apoptotic gene survivin, has already demonstrated efficacy for various cancers in 11 clinical trials. It was shown in vitro that YM155 decreased anti-apoptotic BIRC5/survivin expression resulting in caspase 3/7/8 upregulation and SS cell death [86]. Simultaneous inhibition of Src family kinases and Aurora kinases in the SS model in vivo by the novel inhibitor SU6656 demonstrated broad clinical benefits in cancer therapeutics [87]. Cell cycle regulators, specifically BET inhibitors or the CDK4/6 inhibitor, induced cell cycle arrest and apoptosis [88]. The combination of the mTOR inhibitor ridaforolimus and the epigenetic modifier vorinostat demonstrated in vitro synergism in SS [89]. Another approach to accelerating the activity of epigenetic modulators is to combine HDAC inhibitors with the ERK pathway and heparanase inhibitors [90]. Anlotinib, a new tyrosine kinase inhibitor for oral administration, with primary targets in vasculogenesis and angiogenesis, significantly suppressed synovial sarcoma proliferation in patient-derived xenograft models and in cell lines [89]. Wnt inhibitors, such as NCB-0846, were used in four SS cell lines and a mouse xenograft model induced apoptosis in SS cell lines [91]. Retrospective studies of SS specimens revealed the potential efficacy of the VEGFR-targeted molecule apatinib for advanced SS treatment [92]. An interesting approach was described in the first-in-human (FIH) phase I clinical trial of the monoclonal antibody (OTSA-101) targeting FZD10, the component of the Wnt/β-catenin signaling pathway. However, no objective response was observed [93].

## 5. Conclusions

In this work, we summarized our understanding of the genetic and molecular heterogeneity of synovial sarcoma, an uncommon type of malignant tumor with a mesenchymal origin, and the current and developing approaches to the treatment of this cancer type. Unfortunately, there is a very limited range of effective therapies for synovial sarcoma; treatment is so far confined to surgery, radiotherapy and standard chemotherapy with common cytostatics, and several receptor tyrosine kinase inhibitors (sorafenib, pazopanib and bevacizumab). Innovative approaches include immune-based therapies (NY-ESO-1, MAGE4, PRAME), HDAC inhibitors, BRD disruptors and modifiers of developmental pathways (Notch, Hedgehog, and Wnt/β-catenin pathways). These findings may have the potential to influence sarcoma treatment combined with improved diagnostic and prognostic techniques in vitro, in vivo and ex vivo, re-evaluation of previous paradigms and additional investigation of molecular fingerprints. Considering that the variation of the pathways and genes affected in all SS cases is considerable, simple tests predicting chemoresistance at the early stage of treatment are being developed.

## Figures and Tables

**Table 1:** Main interactors of SSX proteins.

Gene Abbreviation	Description	References
**Regulation of Transcription**		
FUBP3	Far upstream element (FUSE) binding protein 3	[52]
ZSCAN1	Zinc finger and SCAN domain containing 1	
ZBTB25	Zinc finger and BTB domain containing 25	
ZBTB3	Zinc finger and BTB domain containing 3	
PCBD2	Pterin-4 alpha-carbinolamine dehydratase/dimerization cofactor of hepatocyte nuclear factor 1 alpha (TCF1) 2	
NFE2	Nuclear factor (erythroid-derived 2)	
ZNF496	Zinc finger protein 496	
**PcG Protein, the Transcriptional Repressor**		
BMI1	BMI1 polycomb ring finger oncogene	[52]
RING1A	Ring finger protein 1	
RING1B (RNF2)	Ring finger protein 2	
**Transcription Factor**		
LHX4	LIM homeobox 4	[53]
DDIT3 (CHOP)	DNA-damage-inducible transcript 3	[52]
HIF1A	Hypoxia-inducible factor 1, alpha subunit	
PAX9	Paired box 9	
XBP1	X-box binding protein 1	
LZTR1	Leucine-zipper-like transcription regulator 1	
**Protein Transport**		
RAB3IP	RAB3A interacting protein (rabin3)	[52]
**Cell Adhesion**		
SSX2IP	Synovial sarcoma, X breakpoint 2 interacting protein	[52]

**Table 2:** Main clinical trials of SS therapy.

Antigen	Trial Number
**Engineered Immune Cell Adoptive Transfer**	
NY-ESO-1	NCT00670748; NCT01343043; NCT03967223; NCT03399448; NCT04526509; NCT02650986; NCT02457650; NCT01477021; NCT02059850; NCT03450122; NCT03250325; NCT05296564; NCT03520959; NCT02869217
MAGE-A4	NCT04939701; NCT04939701; NCT04044768; NCT03132922
PRAME	NCT03686124
**Immune Checkpoint Inhibitors**	
Anti-PD1	NCT03063632; NCT04356872
Anti-CTLA/anti-PD-L1	NCT02815995
**Receptor Tyrosine Kinase Inhibitors**	
Anlotinib	NCT03016819
**Epigenetic Modifiers**	
HDAC inhibitors (romidepsin, entinostat, vorinostat)	NCT00112463; NCT01136499; NCT01879085; NCT00937495
EZH2 inhibitors	NCT02601937; NCT02875548; NCT02601950; NCT03874455
BDR9 degraders	NCT04965753; NCT05355753

## Data Availability

The data for this study were collected from online resources only.

## Abbreviations

Akt	AKT serine/threonine kinase
APC	Adenomatous polyposis coli
ATR	Ataxia telangiectasia and Rad3 related
AXIN2	axis inhibition protein 2
BCL2	B-Cell lymphoma 2
BMI1	BMI1 proto-oncogene, Polycomb ring finger
BRD	Bromodomain
cBAF	Canonical BRG1/BRM-associated factor
CDCA2	Cell division cycle protein A2
CDC25A	Cell division cycle protein 25A
CDK	Cyclin-dependent kinase
CTA	Cancer/testis antigen
CTLA-4	Cytotoxic T-lymphocyte-associated protein 4
CTNNB1	Catenin beta 1
CXCR4	C-X-C motif chemokine receptor 4
DDIT3	DNA-damage-inducible transcript 3
DDR	Discoidin domain receptor
DKK	Dickkopf WNT signaling pathway inhibitor
EGF	Epidermal growth factor
EGFR	Epidermal growth factor receptor
EPHB4	EPH receptor B4
Etv	ETS-related transcription factor
EZH2	Enhancer of Zeste 2
EWS	Ewing sarcoma protein
FGF	Fibroblast growth factor
FGFR	Fibroblast growth factor receptor
FOXM1	Forkhead box protein M1
FUBP3	Far upstream element (FUSE) binding protein 3
FZD10	Frizzled class receptor 10
GEO	Gene Expression Omnibus
GSK3β	Glycogen synthase kinase-3 beta
HDAC	Histone deacetylase
HGF	Hepatocyte growth factor
HIF1A	Hypoxia-inducible factor 1, alpha subunit
IGF	Insulin-like growth factor
IGFR	Insulin-like growth factor
IGFBP	Insulin-like growth factor binding protein
INI1	SWI/SNF related, matrix associated, actin dependent regulator of chromatin, subfamily b, member 1
KDM2B	Lysine-specific demethylase 2B
KIF14	Kinesin family member 14
KLHL34	Kelch-like family member 34
LEF1	Lymphoid enhancer-binding factor 1
LHX4	LIM homeobox 4
LZTR1	Leucine-zipper-like transcription regulator 1
MDM2	Murine double minute 2
miRNA	MicroRNA
MAGE-A4	Melanoma-associated antigen 4
MAPK	Mitogen-activated protein kinase
MUM1L1	Melanoma-associated antigen (mutated) 1-like 1
MMP	Matrix metalloproteinase
MPNST	Malignant peripheral nerve sheath tumor
mTOR	Mammalian target of rapamycin
NFE2	Nuclear factor (erythroid-derived 2)
NGDN	Neuroguidin
NKX3.1	NK3 homeobox 1
NY-ESO-1	New York esophageal squamous cell carcinoma 1
OSMR	Oncostatin M
PAX7	Paired box 7
PAX9	Paired box 9
PCBD2	Pterin-4 alpha-carbinolamine dehydratase/dimerization cofactor of hepatocyte nuclear factor 1 alpha (TCF1) 2
PD-1	Programmed cell death protein 1
PD-L1	Programmed death ligand 1
PDGF	Platelet-derived growth factor
PDGFR	Platelet-derived growth factor receptor
PI3K	Phosphatidylinositol 3-kinase
PNET	Primary central nervous system (CNS) tumors
PRAME	Preferentially expressed antigen in melanoma
PRC	Polycomb repressive complex
PTCH1	Protein patched homolog 1
PTEN	Phosphatase and tensin homolog
RAB3IP	RAB3A interacting protein (rabin3)
RASAL	RAS protein activator like 1
RING	Ring finger protein 1
SMARCB1	SWI/SNF-related matrix-associated actin-dependent regulator of chromatin subfamily B member 1
SMO	Smoothened
SOX2	SRY-Box transcription factor 2
SS	Synovial sarcoma
STS	Soft tissue sarcoma
SWI/SNF	Switch/sucrose non-fermentable
TCF	Transcription factor 7
TCR	T-cell receptor
TEAD	TEA domain family members
TGF	Transforming growth factor
TLE1	TLE family member 1, transcriptional corepressor
T-TCR	Targeted T-cell receptor gene therapy
TRPS1	Transcriptional repressor GATA binding 1
TrxG	Trithorax-group proteins
VEGFR	Vascular endothelial growth factor receptor
WHO	World Health Organization
WNT5A	Wnt family member 5A
XBP1	X-box binding protein 1
ZBTB25	Zinc finger and BTB domain containing 25
ZBTB3	Zinc finger and BTB domain containing 3
ZIC2	Zic family member 2
ZNF496	Zinc finger protein 496
ZSCAN1	Zinc finger and SCAN domain containing 1
